# A Boosted Minimum Cross Entropy Thresholding for Medical Images Segmentation Based on Heterogeneous Mean Filters Approaches

**DOI:** 10.3390/jimaging8020043

**Published:** 2022-02-11

**Authors:** Walaa Ali H. Jumiawi, Ali El-Zaart

**Affiliations:** Department of Mathematics and Computer Science, Faculty of Science, Beirut Arab University, Beirut 11072809, Lebanon; elzaart@bau.edu.lb

**Keywords:** images segmentation, skin lesion, MRI Alzheimer, MRI brain tumor, improving minimum cross entropy thresholding, heterogeneous mean filters

## Abstract

Computer vision plays an important role in the accurate foreground detection of medical images. Diagnosing diseases in their early stages has effective life-saving potential, and this is every physician’s goal. There is a positive relationship between improving image segmentation methods and precise diagnosis in medical images. This relation provides a profound indication for feature extraction in a segmented image, such that an accurate separation occurs between the foreground and the background. There are many thresholding-based segmentation methods found under the pure image processing approach. Minimum cross entropy thresholding (MCET) is one of the frequently used mean-based thresholding methods for medical image segmentation. In this paper, the aim was to boost the efficiency of MCET, based on heterogeneous mean filter approaches. The proposed model estimates an optimized mean by excluding the negative influence of noise, local outliers, and gray intensity levels; thus, obtaining new mean values for the MCET’s objective function. The proposed model was examined compared to the original and related methods, using three types of medical image dataset. It was able to show accurate results based on the performance measures, using the benchmark of unsupervised and supervised evaluation.

## 1. Introduction

Computer-aided diagnosis systems (CAD) have significant potential for diagnosing fatal diseases and observing their early stages. Evolving image segmentation methods can provide valuable data for diagnosing medical images and accurately detecting the infected regions. Segmentation methods have an important impact on providing a reliable diagnosis with CAD systems. Improving image segmentation methods tends to maximize the accurate detection for the required objects, such as the infected regions in various types of medical images, e.g., dermoscopic skin lesions, MRI of Alzheimer’s, or MRI of brain tumors. Such images represent diseases that rapidly increase over time. The rates of melanoma have increased rapidly over the past few decades. About 132,000 new cases of melanoma are diagnosed each year according to the World Health Organization [1]. Accurate segmentation tries to separate the lesion from the skin region, to observe the early symmetric shape [2,3]. Early diagnosis of this cancerous disease is a very important process, to enucleate rapidly. Alzheimer’s disease (AD) is a gradual neurologic disease in the brain that produces irreversible loss of neurons. AD leads to tissue loss throughout the brain, which shrinks the brain volume. The number of affected people has been increasing every decade, within the next two decades, one out of 85 persons will have AD [4,5]. CAD systems can help neurologists to discover the early stage of AD. Medical resonance imaging (MRI) has been confirmed as very useful in this task [6]. Technically, a CAD system uses MRI to display a useful comparison between normal and abnormal neurons using the analysis of certain characteristics in brain images, and, thus, determine the appearance of atrophied neurons [7]. Moreover, MRI brain tumor segmentation is a hard task for many reasons, such as the non-homogeneous intensities around the tumor, the presence of background noise, the complicated shapes and the fuzzy boundaries, and the low contrast with neighboring brain tissues [8,9].

In general, image thresholding tries to find the best point to split heterogeneous regions [10]. The optimal threshold is the value that represents the highest degree of separation for the intensity levels in a given image. In medical image segmentation, the optimal threshold is the value that helps to indicate the stage of the infected foreground; this is based on the discontinuity and continuity change ratio in intensity levels [11,12]. There are many thresholding methods presented in the literature. In this paper, the aim is to focus on improving the mean-based thresholding method, minimum cross entropy thresholding (MECT), which is one of the most popular and frequently used medical images segmentation methods.

The original MCET method relies on the classical mean approach using the definition of Gaussian distribution [13,14]. The presence of noise, local outliers, and gray levels in image regions are the main challenges when estimating the mean value. According to the objective function of MCET, the optimal threshold relies on the mean value, and enhancing the mean computation process can affect the optimal threshold [15]. Mean filter approaches, such as geometric, harmonic, contra-harmonic, and alpha-trim were proposed to be used heterogeneously for mean estimation; the aim being to exclude the undesired parts, such as noise and outliers from the mean value. The proposed heterogeneous model is a dedicated eliminator and handles the positional presence of the undesired parts, e.g., salt noise present at the end of the image histogram, which belongs to the object region. The same applies for pepper noise present at the beginning of the histogram, which belongs to the background region; and this sample state has many forms that require two different filters to exclude the impact of noise on each region during the mean computation process. Thus, the noise-free mean values for MCET can provide a boosted efficiency for image segmentation. The main contributions of this research are the following:

- Boosting the segmentation efficiency of MCET for early detection of infected foreground in different types of medical images.
- Developing an image segmentation model that contributes to computer-aided-diagnosis (CAD) for precise detection.
- Handling the challenge of noise and outliers, by excluding them from the mean computation process; since the enhanced mean value may positively impact the efficiency of the MCET’s objective function.
- Implementing an inclusive segmentation algorithm for various intensity distributions for a given image.

In this paper, minimum cross entropy thresholding was improved based on the heterogeneous mean filters approach. The rest of the paper is divided as follows. Related work is stated in Section 2. The proposed method is described and explained in Section 3. The performance measures are stated in Section 4. The discussion is explained in Section 5. Finally, the conclusion and future work are detailed in Section 6.

## 2. Related Work

Image segmentation is an important process for the CAD system to detect various types of objects in medical images. Boosting segmentation efficiency provides reliable data that reflect the accurate object detection. Such improvements could have a profound effect on the CAD system and increases its reliability. Nonetheless, medical image segmentation has significant differences that remain a difficult task.

Several thresholding-based segmentation methods have been presented in the literature. Some methods were dedicated to enhancing the accuracy of skin lesion detection [2,14,15,16,17]. Minimum cross-entropy thresholding was proposed by Li et al. [14] to improve the segmentation process, by obtaining the optimal threshold from the optimized entropy. This technique considered one of the most well-known entropy-based thresholding methods. It tries to minimize the variance between two class entropies based on the mean value in each region, as shown in its objective function in Equation (1), which represent the main process for finding the optimal threshold t* after minimizing *n* (*t*).

(1) $$n\left(t\right)=-\sum_{i=1}^{t}\;i\times h\left(i\right)\times \log\left(\mu_{1}^{}\left(t\right)\right)-\sum_{i=t+1}^{L}\;i\times h\left(i\right)\times \log\left(\mu_{2}^{}\left(t\right)\right)$$

where *h*(*i*) refers to the histogram of the grey level *i* in the range [1, *L*]. The regions of the image are considered as two Gaussian distributions, such that the value of $\mu_{1}$(*t*) and $\mu_{2}$(*t*) are estimated in Equations (2) and (3).

(2) $$\;\;\;\;\mu_{1}^{}\left(t\right)=\frac{\;\;\sum_{i=1}^{t}i\times h\left(i\right)}{\sum_{i=1}^{t}h\left(i\right)}\;$$

(3) $$\;\mu_{2}^{}\left(t\right)=\frac{\;\sum_{i=t+1}^{L}i\times h\left(i\right)}{\sum_{i=t+1}^{L}h\left(i\right)}$$

The mean estimator technique of this method is based on Gaussian distribution. However, this type of distribution is often effective for symmetric distribution, but not for the other cases of asymmetric distribution, and asymmetric intensities could have some contradicted parts that affect the mean computation. Nonetheless, the process of mean computation is considered a classical or arithmetic mean in the Gaussian approach; thus, the mean value in most cases can include impacting parts, e.g., the presence of noise, local outliers, and gray areas; thus, it can impact the calculation in Equation (1), according to the direct relationship between the mean value and the objective function.

This method added new possibilities for improvements in the literature, as a solution for difficult image segmentation cases. Chakraborty et al. [16] proposed a particle swarm optimization (PSO) based minimum cross entropy. This method was tested for image segmentation, to show its convergence rate. minimum cross entropy was also improved using Gamma distribution to optimize the final threshold [17]. Moreover, it has been developed using hybrid cross entropy thresholding using Gaussian and Gamma distributions for skin lesion segmentation [2]. The method was also improved for selecting multi-level threshold values, using an improved human mental search algorithm [18]. In addition, it was proposed for color image segmentation based on an exchange market algorithm [19]. Another improvement of this method was proposed based on homogeneous mean filter approaches [15]. This improvement tested multiple types of medical images, to show the impact of the enhanced mean value on the optimal threshold and its positive impact on the segmentation results. However, there are some gaps that need to be filled in this approach, where homogeneous forms try to use one filter for two mean values in two different regions.

As stated in the literature, most of the proposed methods focused on the distribution issue, without taking into account the impact of the mean value on the mean-based MCET, especially when noise, outliers, or gray levels become influential parts within the mean computation, since this original method is based on the classical mean, which includes all the overall intensity levels. MCET was improved based on homogeneous mean filter approaches [15], this improvement proposed excluding certain parts from the total stated issues. It relies on the role of mean filters in homogenous form, without taking into account the combination and the positions of noise in different regions. In this paper, the heterogeneous mean filter approach tries to handle the contradicted intensity levels inside the mean values of each region. This model intends to provide a different and an enhanced mean value for MCET, in order to boost its efficiency. The main aim is to find noise-free mean values; as well as, to evolve the method based on dedicated mean filters in heterogeneous form. The strength of the proposed methods compared to the related methods lies in the impact of the enhanced mean, as shown in Table 1.

## 3. Material and Methods

As stated in the introduction and related work, most of the segmentation techniques have evolved without taking into account the relationship between the mean value and the main objective function of MCET in Equation (1). The literature MCET based on a homogenous mean filter approach indicates one form of improvement, with some noticeable gaps that need to be filled; this is when the mean values are estimated by choosing the same filtering approach for each test case, since some filters are only compatible with a certain type of noise, but not for different regions [15]. In this paper, the gap in the related homogeneous mean-MCET was filled by applying mean filters heterogeneously; such that each mean value was estimated using a mean filter approach, which is different from one that estimates the other mean value.

### 3.1. Dataset Materials

Three types of medical images were used for segmentation in this work (Dermoscopic skin lesion, MRI Alzheimer, and MRI brain tumor). Dermoscopic skin lesions is a public archive dataset; it was acquired from the International Symposium on Biomedical Imaging (ISBI 2016) challenge [20] and Pedro Hispano Hospital (PH2) corpora [21]. In addition, the OASIS MRI Alzheimer disease and MRI brain tumor images were used for segmentation, to perform the experiments on a wide range of images, and each image was examined for segmentation 32 times, while each test case was constructed based on a combination of the proposed heterogeneous mean filter model, as shown in Table 2. On the other hand, there were 21 test cases in the related homogenous approach.

### 3.2. Proposed Mean Filter Approaches

**Figure 1:**
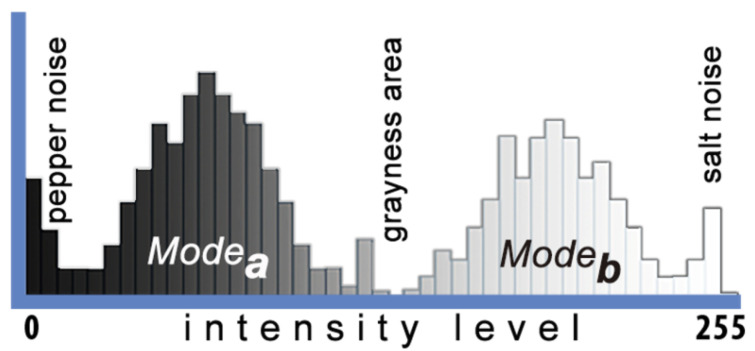
Visualization of the impact of intensity levels in an image histogram.

Filtering approaches such as geometric, harmonic, contra-harmonic, and alpha trim were proposed in literature as noise reduction filters under a pure image processing approach [10]. They were adapted for the histogram version as mean estimators for MCET [15], where both $\mu_{1}$(*t*) and $\mu_{2}$(*t*) were estimated using the same mean filter approach. The use of homogeneous approaches was able to exclude some undesired parts from the mean value to improve MCET. However, some cases faced overlapping issues. In general, the presence of salt noise was located in the highest intensity levels and the pepper noise on the opposite side, as shown in Figure 1. For example, if a harmonic filter used, it will enhance the mean value, with some limitations, e.g., it excludes the salt noise, but it is not suitable for pepper noise in the first region; similarly, when using a contra-harmonic filter with positive and negative values of Q.

**Figure 2:**
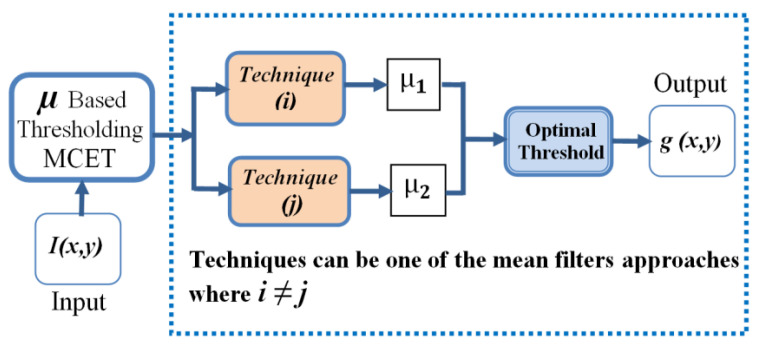
Proposed thresholding framework, where $\mu_{1}$ is estimated using technique (*i*) and $\mu_{2}$ is estimated using technique (*j*) and *i* ≠ *j*.

In this paper, the proposed heterogeneous filter approaches were used as dedicated estimators, as shown in Figure 2. This form can handle different cases of noise distribution. The reported mean filter approaches have effective roles in image processing and noise reduction. The main concept of these approaches is to deal with masks or windows of pixels for mean estimation [10]. They were adapted to deal with image regions for mean estimation [15], by considering the histogram modes as a sorted vector of pixels, as follows:

#### 3.2.1. Classical or Arithmetic Mean Filter

This is the simplest mean filter; it computes the average of the overall values in a given region or mode, as shown in Equation (4).

(4) $$\mu_{a}^{}\left(t\right)=\frac{1}{Length_{a}\;}\sum_{i\in Mode_{a}}^{}Mode_{a}\left(i\right)$$

where $\mu_{a}$(*t*) is the mean value of $mode_{a}$, as stated in Figure 1. Similarly for $\mu_{b}$(*t*) and $mode_{b}$.

#### 3.2.2. Geometric Mean Filter

A geometric mean filter achieves smoothing comparable to the arithmetic mean filter, as shown in Equation (5).

(5) $$\mu_{a}^{}\left(t\right)={\left[\Pi_{i\in Mode_{a}}Mode_{a}\left(i\right)\right]}^{\frac{1}{\;\;Length_{a}}}$$

#### 3.2.3. Harmonic Mean Filter

This filter intends to enhance the mean value, by excluding salt noise, but not for pepper noise. It also works with Gaussian noise, as shown in Equation (6).

(6) $$\mu_{a}^{}\left(t\right)=\frac{Length_{a}}{\sum_{i\in Mode_{a}}^{}\;\;\;\frac{1}{Mode_{a}\left(i\right)}}\;$$

#### 3.2.4. Contra-Harmonic Mean Filter

This filter is used to exclude salt and pepper noise in the image. Depending on its order of *Q* value, the positive values of the order tend to exclude pepper noise, and negative values of its order tend to exclude salt noise, but not both noises; contra-harmonic also reduces to the classical arithmetic mean if its order equals 0, and reduces to a harmonic mean filter if its order equals −1, as shown in Equation (7).

(7) $$\mu_{a}^{}\left(t\right)=\frac{\;\;\;\;\sum_{i\in Mode_{a}}^{}Mode_{a}{\left(i\right)}^{Q+1}}{\sum_{i\in Mode_{a}}^{}Mode_{a}{\left(i\right)}^{Q}}\;$$

#### 3.2.5. Alpha-Trim Mean Filter

This filter can handle multiple types of noise, e.g., a combination of salt and pepper, and also Gaussian noise. This filter could provide a better mean value for asymmetric pixel distribution, depending on the trim value *d*, as shown in Equation (8).

(8) $$\mu_{a}^{}\left(t\right)=\frac{1}{Length_{a}-d}\sum_{i\in Mode_{a}}^{}Mode_{a_{r}}\left(i\right)$$

Both modes represent the regions in the given image histogram, and they have a length that refers to the length of their vectors, such that $length_{a}$ refers to the length of the vector of $mode_{a}$, where $mode_{a}\left(i\right)$ represents the intensity level *i* that belong to $mode_{a}$, and $mode_{a_{r}}\left(i\right)$ in alpha trim filter represents the remaining intensity level *i* that belongs to $mode_{a}$; similarly, for $mode_{b}$.

### 3.3. Proposed Heterogeneous Model

**Figure 3:**
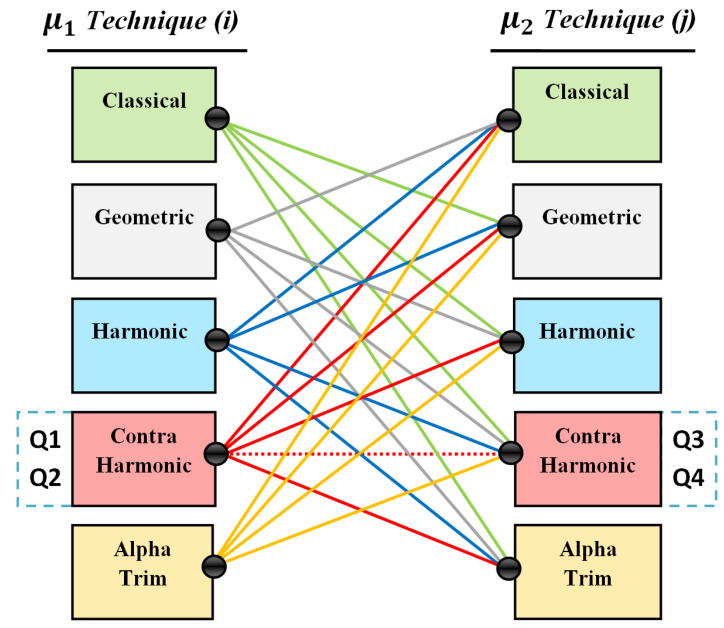
Proposed heterogeneous mean filter model for Figure 2, where *i* ≠ *j*.

The heterogeneous approach is the model where the first mean value in $Mode_{a}$ and second mean value in $Mode_{b}$ should not use the same filtering approach, as stated in Figure 2 and Figure 3, where the approach that was used to estimate $\mu_{1}$(*t*) is not used to estimate $\mu_{2}$(*t*), as explained in Figure 3, such that, technique (*i*) and technique (*j*) are always heterogeneous.

However, there are some exceptions in the heterogeneous model, such that the same filtering approach can be used for the both regions, but in a heterogeneous input, e.g., a contra-harmonic filter can used homogeneously for each region with different inputs of order Q, as shown in Figure 3, where the positive values Q1 and Q2 can act as a heterogeneous approach with the negative values Q3 and Q4. In addition, the alpha trim filter was selected based on the best results from the related works based on the homogenous model [15], where d/2 = 55 was indicated as the best trim value for mean estimation with MCET.

The proposed model relies on five mean filter approaches, including usage of the classical mean; this includes the convergence with the input from the original method. The selected parameter for the alpha trim mean filter keeps one input, and the contra-harmonic filter was used with two positive and two negative values of Q, where positive values of Q were selected to estimate the first mean, and negative values were selected for estimating the second mean; this is depending on the role of this filter for noise reduction. The overall heterogeneous test cases in this paper numbered 32 cases.

The main aim of the proposed heterogeneous model is to handle various kinds of noise in image regions when estimating the mean values for MCET, such that it handles the contradicted presence of the noise in each region in a given image using the same filter, which is the main drawback in the related works when using a homogeneous approach.

### 3.4. Proposed Algorithm

The time complexity to find the value t* is $O(L_{}^{2})$, using a homogenous distribution [2]. However, searching for the optimal threshold can be time consuming with the heterogeneous model. For x thresholding model, the time complexity is $O(L_{}^{x+1})$. In order to perform the algorithm in less time, parallel processing can provide a sufficient resource for this issue. The proposed algorithm was implemented using the parallel processing toolbox in Matlab 2019. In this way, the overall computational processes can be performed with a better performance than with sequential processing, as shown in Table 3.

Algorithm 1 represents a single model using one of the heterogeneous mean estimation inputs, this sequential algorithm should be performed 32 times based on each input in Table 2. However, modeling segmentation results can be achieved based on the best threshold from the all the heterogeneous inputs. In this paper, the algorithm was implemented using parallel processing, and the performance measures were computed for each threshold in each heterogeneous input. The best threshold is the value that maximizes the performance measures, as shown in Algorithm 2.
**Algorithm 1** Single Input-Sequential ProcessingRead input image *I*(*x*, *y*)Compute the histogram *h*(*i*), *i* = 0, …., 255 for *I*(*x*, *y*)for *t* = 1: 255 do Compute $\mu_{1}$(*t*) using filtering approach ∈ technique (*i*) (see Figure 3).Compute $\mu_{2}$(*t*) using filtering approach ∈ technique (*j*) (see Figure 3)Compute the objective function *n*(*t*) in Equation (4).Find the optimal t* which minimize *n*(*t*).End for.return t*Output image *g*(*x*, *y*).❖For other mean filters approaches:
Compute $\mu_{1}$(*t*) and $\mu_{2}$(*t*) according to their equations (see Table 2)

**Algorithm 2** Multiple Input-Parallel Processing
Read input image *I*(*x*, *y*)Compute the histogram *h*(*s*), *s* = 0, …., 255 for *I*(*x*, *y*) for *t* = 1: 255 do *i* ≠ *j* (see Figure 3)Compute MCET’s objective function *n*(*t*) in Equation (4) base on the following inputs:
Compute $\mu_{1}$(*t*) and $\mu_{2}$(*t*) using the heterogeneous inputs 1, 2, 3 … 32 (see Table 2)
Compute *t*1*, *t*2*, … *t*32* that minimize *n*(*t*) for each heterogeneous inputs.End for.Compute the average sum of the performance measure for each t*.Find the best t* which maximize the performance measure.Return the best t*Output image *g*(*x*, *y*).


## 4. Performance Measure

In this paper, unsupervised and supervised performance measurement was used for evaluation; these measurements were also used to compare the segmentation accuracy between the proposed model and related methods [22,23,24,25,26,27,28].

### 4.1. Unsupervised Evaluation

Using unsupervised evaluation, it was possible to evaluate the segmentation results without any a priori information. This evaluation mainly depends on a statistical approach between the segmented image and the original image. For region segmentation, unsupervised evaluation takes into account the uniformity, region contrast, and inter-region disparity.

#### 4.1.1. Image Uniformity

This measurement helps assess the quality of the thresholding method, since region uniformity is computed based on variance. This measurement was proposed by Levine et al. [22]. The *IU* is defined in Equation (9).

(9) $$IU\left(t\right)=1-\frac{\sigma 1^{2}\left(t\right)-\sigma 2^{2}\left(t\right)}{Z}$$

where *Z* is calculated as shown in Equation (10), and $\sigma 1^{2}\left(t\right)$ and $\sigma 2^{2}\left(t\right)$ are the variances of $mode_{a}$ and $mode_{b}$, respectively, as stated in Figure 1.

(10) $$Z=\frac{{\left(I_{max}-I_{min}\right)}^{2}}{2}$$

where $I_{max}$ and $I_{min}$ are the maximum and the minimum intensity levels.

#### 4.1.2. Region Contrast

This measurement helps to indicate the adjacent regions and check the high contrast; thus, it assesses the quality of segmented results, as shown in Equation (11).

(11) $$RC\left(t\right)=\frac{\left|\mu_{1}^{}\left(t\right)-\mu_{2}^{}\left(t\right)\right|}{\mu_{1}^{}\left(t\right)-\mu_{2}^{}\left(t\right)}$$

where $\mu_{1}\left(t\right)$ and $\mu_{2}\left(t\right)$ are the mean values of the first and the second region in a given image, respectively.

#### 4.1.3. Inter-Region Disparity

This measurement is based on the interior and the external contrast [24], as shown in Equations (12) and (13), respectively, where *c*(*s*,*t*) is the contrast pixels *s* and *t*, as shown in Equation (14).

(12) $$\;\;\;\;\;\;\;\;\;CI\left(i\right)=\frac{1}{A_{i}}\sum_{s=R_{i}}^{}Max\left\{c\left(s,t\right),\;t\in W\left(s\right)\cap R_{i}\right\}$$

(13) $$\;\;\;\;\;\;\;\;\;\;CE\left(i\right)=\frac{1}{l_{i}}\sum_{s=F_{i}}^{}Max\left\{c\left(s,t\right),\;t\in W\left(s\right)\notin R_{i}\right\}$$

(14) $$\;\;\;\;\;\;\;c\left(s,t\right)=\frac{\left|I\left(s\right)-I(t)\right|}{L-1}$$

where *Ii* is the length *Fi* border in the region *Ri*, *Ai* corresponds to the surface of *Ri*, and *W*(*s*) refers to the neighborhood of the pixel *s*. As in the previous Evaluation, the scores in this measurement belong to [0, 1], since the closest to 0 indicates poor segmentation scores, and the closest to 1 indicates good segmentation results, as shown in Equation (15).

(15) $$C\left(R_{i}\right)=\begin{array}{ll} 1-\frac{CI\left(i\right)}{CE\left(i\right)} & \mathrm{if}\;0<CI\left(i\right)<CE\left(i\right)\\ CE\left(i\right) & \mathrm{if}\;CI\left(i\right)=0\\ 0 & \mathrm{otherwise}\; \end{array}\;$$

### 4.2. Supervised Evaluation

**Figure 4:**
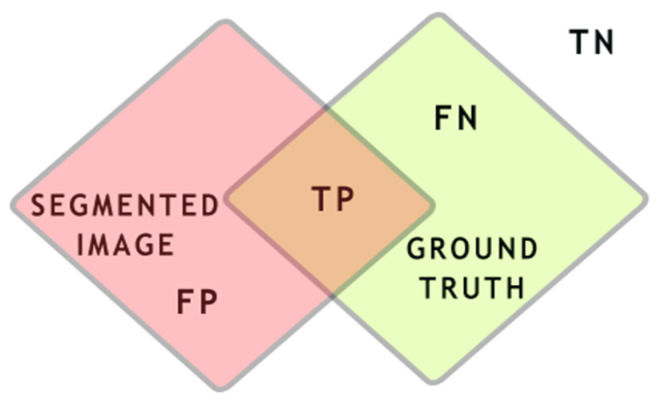
TN represent the pixels that have not been segmented, FN represent the pixels that should appear in the segmented image, TP are the joint segmented pixels, and FP represents the pixels that should not have been segmented.

This measurement is based on a comparison approach between the segmented image and its ground truth. This evaluation is widely used in the literature as a powerful measurement to assess quality scores [25,26,27,28]. The goal is to maximize the true positivity TP of pixels in the segmented image, as explained in Figure 4. Based on these terms, Jaccard index JI, F-score, and the segmentation accuracy were used to evaluate the matching of the pixels between the segmentation results and the ground truths.

The Jaccard index was used to assess the intersection ratio, as shown in Equation (16). F-scores help to assess the probability of the true positive pixels in Equation (17), where precision refers to TP/TP + FP; recall refers to TP/TP + FN, where precision is used to measure the detected actually true pixels; and recall measures the true positivity and the probability that a segmented pixel belongs to the ground truth. Segmentation accuracy is used to find the degree of matching between the pixels from the segmentation result and the ground truths, as shown in Equation (18).

(16) $$\;\;{\mathrm{Jaccard}}_{\mathrm{index}}=\frac{\mathrm{TP}}{\mathrm{TP}+\mathrm{FP}+\mathrm{FN}}\;$$

(17) $${\;F}_{\mathrm{Score}}=2*\frac{\mathrm{Precision}*\mathrm{Recall}}{\mathrm{Precision}+\mathrm{Recall}}$$

(18) $$\;\mathrm{Accuracy}=\frac{\mathrm{TP}+\mathrm{TN}}{\mathrm{FN}+\mathrm{FP}+\mathrm{TP}+\mathrm{TN}}$$

The average value of the supervised and the unsupervised evaluation scores were computed for performance comparison, as well as to show convergence rate for various measurements on the segmentation outputs.

### 4.3. Modeling the Accurate Segmentation

Heterogeneous mean filter approaches were applied as a mean estimation model for the MCET. In this method, the threshold value minimizes the objective function for each input in Table 2. Computing the optimal threshold every time the heterogeneous input is applied, makes the segmentation problem a nondeterministic polynomial (NP)-Hard optimization problem [2]. Minimizing the threshold value *t* is the process that indicates the optimal threshold t* for the image that needs to be accurately segmented; at the same time the accurate segmentation aims to maximize the evaluation metrics as an accuracy indicator, as shown in Equation (19)

(19) Maximize Evaluation( Unsupervised, Supervised )

where unsupervised (*IU*(*t*), *RC*(*t*), *C*(*Ri*)), and supervised evaluations (${\mathrm{Jaccard}}_{\mathrm{index}},{\;F}_{\mathrm{Score}},\;\mathrm{Acuuracy})$ ∈ [0, 1]. This maximization indicates the best threshold among all the heterogeneous inputs to the MCET, as stated in Section 3, Algorithm 2.

## 5. Discussion

In this paper, we examined three types of medical images for segmentation. The dermoscopic skin lesion and MRI brain tumor were applied with their corresponding ground truths to compute a supervised evaluation that relies on the ground truth, while the unsupervised evaluation was computed for these two datasets as well. Alzheimer images were applied for segmentation without ground truths, and segmented results were evaluated using unsupervised evaluation. Based on the heterogeneous inputs from Figure 3 and Table 2, the proposed thresholding model used five mean filter approaches, where $\mu_{1}^{}\left(t\right)$ was estimated using a filtering approach that was different from the approach that estimated $\mu_{2}^{}\left(t\right)$. The computed mean values were applied on MCET to check their impacts. The proposed heterogeneous approach includes special cases from the related homogenous approahes; e.g., a contra-harmonic mean filter was applied homogeneously with heterogeneous input of Q values −1.5, −0.5, +0.5, +1.5; these values act as a dedicated input to exclude noise combinations in different regions, where the positive value of Q is suitable for the first mean via its aim of excluding the pepper noise, and a negative value of Q is suitable for the second mean via its aim of excluding the salt noise. In addition, the trim value in the alpha trim filter d/2 = 55 was selected as the best trim for mean estimation with MCET [15].

**Figure 5:**
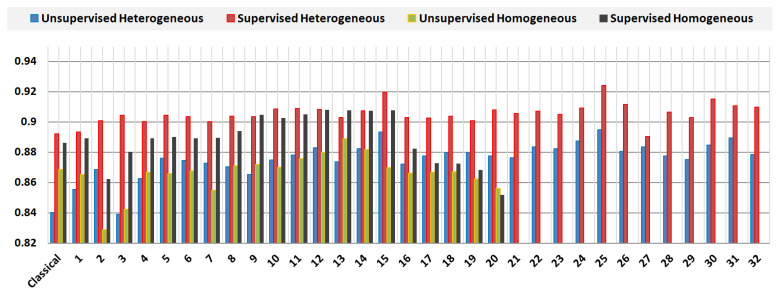
Supervised and unsupervised performance measure comparing MCET using the classical mean, homogeneous mean estimation approach, and the heterogeneous mean estimation approach.

The first input in Table 2 refers to the original Gaussian-based MCET that has a steady classical mean. The rest of the inputs refer to the related homogenous mean-based MCET and the proposed heterogeneous mean-based MCET. The comparison of experiments was examined using the average value of the unsupervised and the supervised evaluation score. The proposed heterogeneous model was able to produce effective results over the related methods; depending on the patterns of the evaluation in Figure 5. It can be noticed that the best segmentation results appeared in test case number 25; this is $\mu_{1}^{}\left(t\right)$ and $\mu_{2}^{}\left(t\right)$ were estimated using the contra-harmonic mean filter with heterogeneous Q = +0.5 and −1.5, respectively. The impact was noticeable also in the test case number 15 compared with the related homogeneous approach; this is $\mu_{1}^{}\left(t\right)$ was estimated using the harmonic mean filter and $\mu_{2}^{}\left(t\right)$ was estimated using an alpha trim mean filter with d/2 = 55. The proposed model showed that it has the ability to fill the gaps related to noise combinations in different regions that were found in the related homogeneous mean-based MCET.

The test cases 15, 20, 24, 25, 26, 30, and 31 showed increased efficiency, based on the performance measure. This improvement indicates the positive impact from the enhanced mean and the segmentation efficiency. Although the related homogeneous mean-based MCET had some improvements in test cases number 12, 13, and 14, it still had a limitation regarding the usage of the same filter for each region. While, the proposed method showed that combining different mean filters was able to boost the segmentation efficiency, as shown in inputs number 15, 25, and 30, the gaps were almost filled in these test cases when filtering approaches combined together heterogeneously. Moreover, the proposed model shows that the contra harmonic approach has a positive impact on mean computation when using positive and negative values of Q heterogeneously for each region in an image histogram.

As a comparison, and as shown in Table 4, the proposed heterogeneous model showed a 3.75% increase in rate based on the average evaluation scores compared with the original MCET efficiency. This rate indicates an objective boost in segmentation accuracy, and an increase in the true positivity rate for the segmented image compared with the homogeneous model. While the homogeneous model showed about a 2.80% rate increase compared to the original method.

**Figure 6:**
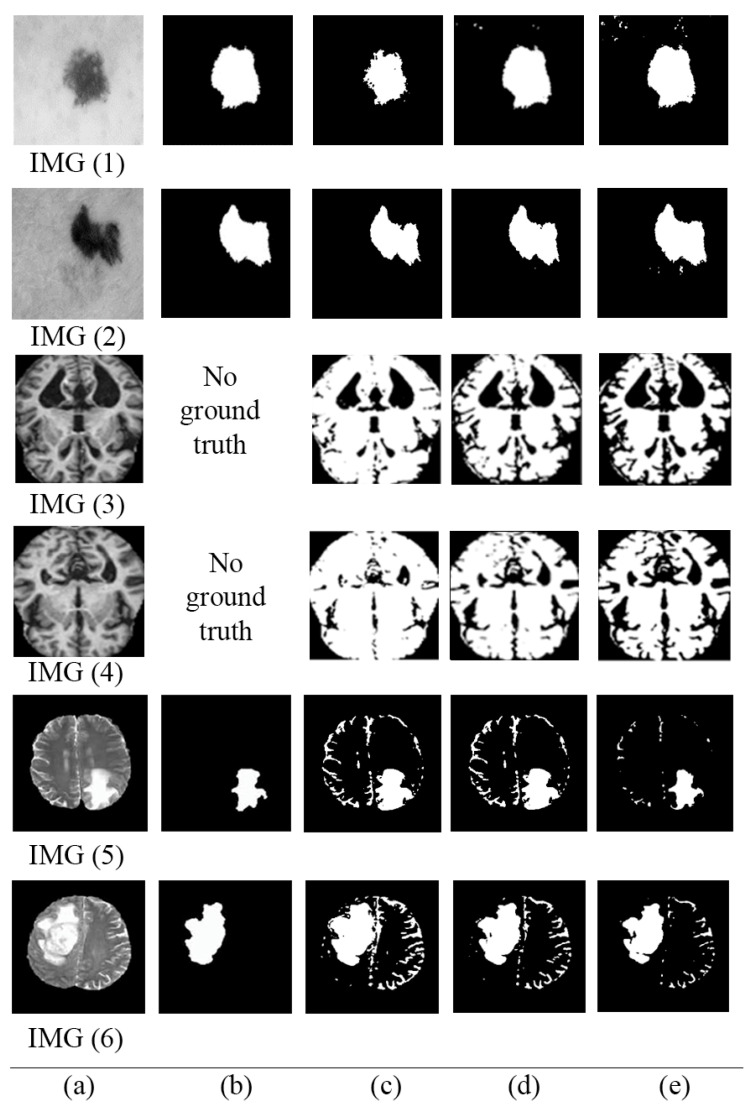
Samples of segmented images (**a**) original images, (**b**) ground truths, (**c**) segmented images using the original MECT, (**d**) segmented images using MCET based on homogeneous mean filter approaches, (**e**) segmented images using the proposed MCET based on heterogeneous mean filter approaches.

Figure 6 shows randomly selected images and their corresponding segmentation results. Figure 6a–e shows original images, ground truths, and segmented images using the original MECT, segmented images using MCET based on homogeneous mean filters approaches, and segmented images using the proposed MCET based on heterogeneous mean filter approaches, respectively. Table 5 shows the performance measured using supervised and unsupervised evaluation for segmented images in Figure 6.

## 6. Conclusions and Future Work

This paper presents an improvement of minimum cross entropy thresholding MCET for medical image segmentation. The improvement was based on a heterogeneous model of mean filter approaches. The aim was to estimate noise-free mean values for a MCET objective function, and positively impact segmentation efficiency based on the relation between the mean value and the optimal threshold. The proposed model was evaluated using supervised and unsupervised evaluation approaches as a performance measure. This paper contributes to computer-aided-diagnosis (CAD) for precise detection and accurate foreground segmentation; as well as to overcoming the challenge of the steady classical mean values in the original MCET. In addition, it provides improved performance for image segmentation over the related methods, as it can handle the gaps of noise distribution in image regions.

In future work, this model could be used to examine various types of medical and optical images, so as to extend the contribution to a wide domain of applications for image segmentation. In addition, this segmentation model could be developed using deep learning-based segmentation, as it has an open adaptation of mean estimation for heterogeneous intensity levels in different images.

## Figures and Tables

**Table 1:** Mean impact and effectiveness of the proposed method compared to the related methods.

MCET Methods	Mean Values	Effectiveness
Proposed MCET based on heterogeneous mean	Dedicated Enhancement	Suitable for various intensity distributions, heterogeneous form of mean estimation for different intensity levels in image regions
MCET based on homogeneous mean [15]	Partially enhanced	Limited to single filter role per region, homogeneous form of mean estimation
MCET-hybrid distributions [2]	Steady	Limited to certain mean values, e.g., Gaussian (classical mean) or lognormal definition

**Table 2:** $\mu_{1}$ and $\mu_{2}$ estimation inputs of heterogeneous and homogeneous mean filter approaches for MCET.

Heterogeneous			Homogeneous [15]
$\mu_{1}\;\mathrm{Estimation}$		$\mu_{2}\;\mathrm{Estimation}$	$\mu_{1}$ $\mu_{2}\;\mathrm{Estimation}$
0	Classical	Classical	Classical
1	Classical	Geometric	Harmonic
2	Classical	Harmonic	Geometric
3	Classical	C-Harmonic, Q = −1.5	C-Harmonic, Q = −3.0
4	Classical	C-Harmonic, Q = −0.5	C-Harmonic, Q = −1.5
5	Classical	Alpha Trim, d/2 = 55	C-Harmonic, Q = −0.5
6	Geometric	Classical	C-Harmonic, Q = +0.5
7	Geometric	Harmonic	C-Harmonic, Q = +1.5
8	Geometric	C-Harmonic, Q = −1.5	Alpha Trim, d/2 = 10
9	Geometric	C-Harmonic, Q = −0.5	Alpha Trim, d/2 = 20
10	Geometric	Alpha Trim, d/2 = 55	Alpha Trim, d/2 = 30
11	Harmonic	Classical	Alpha Trim, d/2 = 40
12	Harmonic	Geometric	Alpha Trim, d/2 = 50
13	Harmonic	C-Harmonic, Q = −1.5	Alpha Trim, d/2 = 55
14	Harmonic	C-Harmonic, Q = −0.5	Alpha Trim, d/2 = 60
15	Harmonic	Alpha Trim, d/2 = 55	Alpha Trim, d/2 = 65
16	C-Harmonic, Q = +1.5	Classical	Alpha Trim, d/2 = 70
17	C-Harmonic, Q = +1.5	Geometric	Alpha Trim, d/2 = 80
18	C-Harmonic, Q = +1.5	Harmonic	Alpha Trim, d/2 = 90
19	C-Harmonic, Q = +1.5	C-Harmonic, Q = −1.5	Alpha Trim, d/2 = 100
20	C-Harmonic, Q = +1.5	C-Harmonic, Q = −0.5	Alpha Trim, d/2 = 110
21	C-Harmonic, Q = +1.5	Alpha Trim, d/2 = 55	-
22	C-Harmonic, Q = +0.5	Classical	-
23	C-Harmonic, Q = +0.5	Geometric	-
24	C-Harmonic, Q = +0.5	Harmonic	-
25	C-Harmonic, Q = +0.5	C-Harmonic, Q = −1.5	-
26	C-Harmonic, Q = +0.5	C-Harmonic, Q = −0.5	-
27	C-Harmonic, Q = +0.5	Alpha Trim, d/2 = 55	-
28	Alpha Trim, d/2 = 55	Classical	-
29	Alpha Trim, d/2 = 55	Geometric	-
30	Alpha Trim, d/2 = 55	Harmonic	-
31	Alpha Trim, d/2 = 55	C-Harmonic, Q = −1.5	-
32	Alpha Trim, d/2 = 55	C-Harmonic, Q = −0.5	-

**Table 3:** Time performance of the proposed algorithm when implemented using parallel processing.

Segmented Images		No. of Images	Sequential(s)	Parallel(s)	Speed-Up Gain
1	MRI Alzheimer	100	488.701	298.733	38.87%
2	Skin Lesion	150	595.487	377.518	36.59%
3	MRI Brain	50	401.010	232.741	41.97%

**Table 4:** Overall comparison between the proposed MCET based on heterogeneous mean filter approaches and the related methods.

	Original MCET	MCET Hybrid Distributions [2]	MCET Homogeneous [15]	Proposed MCET Heterogeneous
Avg. Evaluation	0.87790	0.89262	0.90255	0.91098
Performance	-	1.67%	2.80%	3.75%
Best Mean Value	-	Steady	3/20	7/32

**Table 5:** Average performance measures for the selected images in Figure 6, columns (A) refer to supervised evaluation; columns (B) refer to unsupervised evaluation.

Classical			Homogeneous		Heterogeneous	
	A	B	A	B	A	B
IMG (1)	0.88724	0.84533	0.91613	0.88276	0.91824	0.88525
IMG (2)	0.89053	0.85186	0.91105	0.87987	0.92150	0.89816
IMG (3)	-	0.80779	-	0.89926	-	0.91977
IMG(4)	-	0.82581	-	0.90140	-	0.90996
IMG (5)	0.81448	0.80779	0.91800	0.89926	0.93895	0.91977
IMG(6)	0.84033	0.82581	0.91772	0.90140	0.92407	0.90996

## Data Availability

Data presented in this study are available on request.
